# The Effect of Artemether–Lumefantrine Combined with a Single Dose of Primaquine on *Plasmodium falciparum* Gametocyte Clearance and Post-Treatment Infectivity to *Anopheles arabiensis*

**DOI:** 10.3390/tropicalmed11010019

**Published:** 2026-01-08

**Authors:** Awoke Minwuyelet, Delenasaw Yewhalaw, Giulio Petronio Petronio, Roberto Di Marco, Getnet Atenafu

**Affiliations:** 1 Department of Biology, College of Natural and Computational Science, Debre Markos University, Debre Markos P.O. Box 269, Ethiopia; 2 Tropical and Infectious Diseases Research Center, Jimma University, Jimma P.O. Box 5195, Ethiopia; 3 School of Medical Laboratory Sciences, Faculty of Health Sciences, Jimma University, Jimma P.O. Box 378, Ethiopia; 4 Department of Medicine and Health Sciences “Vincenzo Tiberio”, Università degli Studi del Molise, 86100 Campobasso, Italy; 5 Department of Drug and Health Sciences, Università degli Studi di Catania, 95131 Catania, Italy

**Keywords:** *Plasmodium falciparum*, gametocytemia, artemether-lumefantrine, primaquine, infectivity, membrane feeding assays, *Anopheles arabiensis*

## Abstract

Background: Malaria remains a major public health concern in Africa, due to the persistence of *Plasmodium falciparum* gametocytes that sustain transmission post treatment. This study evaluated the effects of artemether–lumefantrine (AL) alone compared with AL combined with a single low-dose of primaquine (SLD-PQ) on gametocyte clearance and infectivity to *Anopheles arabiensis* post treatment. Methods: A prospective cohort and entomological study were conducted from January to September 2025 in Northwest Ethiopia. Ninety-six microscopically confirmed cases of *P. falciparum* gametocytemia mono-infection were proportionally assigned to both treatment groups. Follow-up assessments were conducted on days 3, 7, 14, and 28, and mixed-species infections were assessed using molecular diagnostic assays. Additionally, membrane feeding assays (MFAs) were performed to evaluate mosquito infectivity post treatment. Results: Gametocyte prevalence declined faster with AL + SLD-PQ (15.2% on day 3; 0% by day 7) compared to AL alone (28.9% on day 3: *p* = 0.001; 12.2% by day 7: *p* = 0.033). Higher baseline gametocyte density strongly predicted mosquito infection (95% in high vs. 59% moderate and 33% low). On day 3 post treatment, 28.6% of cases treated with AL only showed confirmed mosquito infection, compared to 6.8% in the AL + SLD-PQ group (*p* = 0.001). By day 7, 7.3% of cases remained infectious in the AL-only group, while none were detected in the AL+ SLD-PQ group (*p* = 0.01). Conclusions: High baseline gametocyte density strongly correlated with increased infectivity. Adding SLD-PQ markedly accelerates gametocyte clearance and completely blocks post-treatment transmission. Submicroscopic gametocytemia contributed to residual transmission in the AL-only group. Incorporation of SLD-PQ alongside AL, in line with WHO recommendations, is advised to enhance post-treatment transmission blocking, with continued surveillance.

## 1. Introduction

Malaria remains a major global health challenge, significantly contributing to illness and death, particularly in sub-Saharan Africa. According to the World Health Organization’s (WHO) malaria report in 2024, nearly 263 million malaria cases were reported, with an incidence of 60.4 cases per 1000 populations at risk. The WHO African region continues to bear the highest burden of the disease, accounting for an estimated 94% of malaria cases worldwide in 2023 [1].

Based on the WHO malaria report 2025, there were an estimated 282 million malaria cases and 610, 000 deaths globally. The highest burden of malaria cases was reported in Nigeria (68,466,000 cases), followed by the Democratic Republic of the Congo (35,175,000 cases), Uganda (13,216,000 cases), and Ethiopia (12,437,000 cases). Alarmingly, the highest increases in malaria-related deaths were also observed in three countries, namely Madagascar (over 4900), Ethiopia (over 3800), and Yemen (over 932), which together accounted for 85% of the rise in deaths from 2023 to 2024 in the world [2]. These trends underscore the fragility of recent gains and the urgent need for strengthened surveillance, diagnosis, and transmission-blocking interventions.

Effective antimalarial treatment is crucial not only for curing the disease but also for interrupting its transmission to *Anopheles* mosquitoes, which is a vital step toward achieving malaria control and eventual elimination [3]. Artemisinin-based combination therapies (ACTs) remain the cornerstone for treating uncomplicated *P. falciparum* malaria and have demonstrated high efficacy across Africa. However, recent evidence points to the emergence of partial artemisinin resistance in East African *P. falciparum* strains, raising concerns about the long-term viability of these regimens [4,5].

Among ACTs such as artesunate–amodiaquine dihydroartemisinin–piperaquine, artesunate–pyronaridine, artesunate–sulfadoxine–pyrimethamine, arterolane–piperaquine, artemisinin–naphthoquine, and artemisinin–piperaquine [6,7], AL is indeed the most widely procured ACT in sub-Saharan Africa. It is also one of the six ACT options recommended by the WHO for the treatment of uncomplicated *P. falciparum* malaria. The widespread adoption of AL is supported by its effectiveness in reducing parasite biomass and alleviating malaria symptoms, as demonstrated in numerous studies across the region [8,9,10].

AL sold under the brand name Coartem^®^ effectively clear asexual blood-stage parasites and reduces subsequent gametocyte carriage by preventing new gametocytogenesis. However, its effectiveness against mature gametocytes, specifically the transmissible sexual forms, is limited and inconsistent [3,11,12]. *P. falciparum* gametocytogenesis progresses through five developmental stages (I-V). Only stage V gametocytes can infect mosquitoes and sustain transmission. When mature gametocytes are present at the time of treatment, they often persist after ACT administration, sometimes at submicroscopic levels undetectable by conventional microscopy. Consequently, patients may remain infectious to mosquitoes even after clinical recovery, which poses a significant challenge for elimination efforts [13].

As global and national malaria programs increasingly focus on elimination, targeting mature gametocytes has become a priority. In this context, combining ACTs such as AL with SLD-PQ, a potent gametocytocidal drug, has been proposed as a promising strategy to enhance transmission-blocking efficacy. Primaquine clears the liver-stage parasites and specifically targets mature gametocytes, reducing their availability to mosquitoes and lowering transmission threat. Since female *Anopheles* mosquitoes are the primary vectors of malaria, such gametocytocidal interventions are critical components of integrated control and better advancing elimination strategies [3,12,14,15].

The addition of primaquine contributes not only to effective case management but also plays a pivotal role in long-term malaria control by interrupting transmission and it has also the potential to slow the selection and spread of drug-resistant parasites [11,16]. However, evidence regarding the transmission-blocking efficacy of primaquine remains mixed, with significant variability reported across studies. These discrepancies often arise from methodological differences, particularly in mosquito feeding experiments like MFAs, which are used to evaluate post-treatment gametocyte infectivity and their ability to initiate infection in mosquitoes [12,15].

Primaquine, an 8-aminoquinoline antimalarial, is known for its gametocytocidal activity against *P. falciparum*, making it a key tool in interrupting malaria transmission [17]. Despite concerns over its hemolytic potential in individuals with glucose-6-phosphate dehydrogenase (G6PD) deficiency, the WHO recommends the use of SLD-PQ without prior G6PD testing in specific transmission settings, given the minimal risk of hemolysis from a single dose and the significant public health benefits associated with reduced transmission [18,19,20].

Similarly, the malaria burden in Ethiopia is increasing continuously and becoming more public health challenges [21,22,23]. The Ethiopian Federal Ministry of Health’s (FMOH) 2022 annual performance report revealed a troubling 66% increase in malaria cases, rising from 904,495 in 2019 to 1,504,405 in 2022 [24]. Data from the WHO also showed that between 1 January and 20 October 2024, Ethiopia reported over 7.3 million malaria cases and 1157 deaths, resulting in a case fatality rate of 0.02%. It requires substantial effort to tackle the challenge.

In addition to its high prevalence, the emergence of chloroquine-resistant *P. falciparum* in Ethiopia prompted a policy shift to sulfadoxine–pyrimethamine (SP); however, resistance to SP also developed rapidly [25,26]. Subsequently, Ethiopia’s malaria treatment policy shifted to artemisinin-based therapy for *P. falciparum* since 2004 [27]. Currently, no artemisinin-resistance-associated mutations have been detected in the Amhara region, although such mutations have been reported in other parts of Ethiopia [26,28]. Since 2018, Ethiopia has also introduced the use of an SLD-PQ for uncomplicated *P. falciparum* cases. It is used as part of routine treatment to further reduce transmission [29]. In line with global eradication efforts, Ethiopia has implemented a national malaria strategic plan for 2021–2025, which has recently been extended to 2030, with a renewed emphasis on achieving malaria elimination in low-transmission areas [22]. Central to malaria transmission in Ethiopia is *An. arabiensis*, a member of the *Anopheles gambiae* complex, known for its widespread distribution and significant ecological adaptability. Numerous entomological surveys conducted across the country consistently identify *An. arabiensis* as the predominant vector species [30,31]. However, the recent introduction and spread of the invasive urban vector *An. stephensi*, now reported in several Ethiopian urban and peri-urban areas, increase the transmission risk in cities and towns and complicate control strategies, reinforcing the case for interventions that shorten the infectious period [32,33,34].

The MFAs remain the standard method for evaluating the transmission-blocking efficacy of antimalarial interventions [35]. Evidence indicates that combining ACT with SLD-PQ markedly reduces gametocyte prevalence and subsequent human-to-mosquito transmission. Specifically, this combination has been shown to significantly diminish the infectiousness of *P. falciparum* to *Anopheles* mosquitoes, as demonstrated by reduced oocyst development in MFA studies [36,37,38].

As part of Ethiopia’s national malaria elimination efforts, routine surveillance of the therapeutic efficacy of AL combined with SLD-PQ is a critical component. The present study was designed to evaluate the effectiveness of AL alone, and in combination with SLD-PQ, for the treatment of uncomplicated *P. falciparum* malaria at the Bichena Primary Hospital and nearby health centers in Northwest Ethiopia. It also assessed the post-treatment transmission potential of *P. falciparum* to *An. Arabiensis*, using MFAs to provide evidence for optimizing elimination strategies in the study area.

## 2. Materials and Methods

### 2.1. Study Area

**Figure 1:**
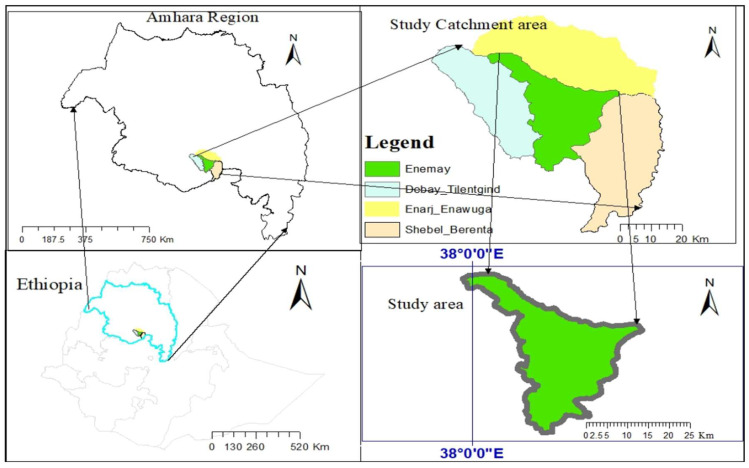
Map of the study area including the catchment of the Bichena Primary Hospital and Enemay District, Amhara Region, Ethiopia.

This study was carried out at the Bichena Primary Hospital and three health centers in the Enemay district of Northwestern Ethiopia, located near the Abay Valley Gorge, approximately 263 km northwest of the capital, Addis Ababa. The hospital serves surrounding districts such as Enarj Enawuga, Shebel Berenta, and Debay Tilatgind, in addition to the Bichena town administration and the wider Enemay District population (Figure 1). The additional study sites included the Bichena Health Center, Woyra Health Center, and Yetmen Health Center, which are located within the catchment of the Bichena Primary Hospital and administratively governed by the Enemay District. Previous studies conducted in the Amhara region have shown that malaria transmission intensity varies by altitude and landscape. Higher transmission intensities are observed in low-altitude areas with suitable vector habitats, while higher-altitude areas experience lower but still unstable transmission [39]. Other studies also reported varying transmission patterns, with some areas experiencing low local transmission despite high importation rates [40]. Even though the specific classification of transmission intensity of the study area requires detailed local epidemiological data, the study area experiences seasonal malaria transmission and provides an essential setting for evaluating malaria treatment efficacy and transmission dynamics. In this malarious setting, *P. falciparum* and *P. vivax*, co-exist throughout the year, with malaria transmission occurring seasonally and peaking from September to November and again from May to July [41,42]. The primary malaria vector identified in the region is *An. arabiensis* [43,44]. Antimalarial drug resistance, particularly to chloroquine and sulfadoxine–pyrimethamine (SP), is high in *P. falciparum*. Artemisinin resistance has been documented in various regions of East Africa, including Ethiopia, with mutations in the Pf Kelch gene being a significant factor [45]; however, specific data on the study area or nearby is limited. Although ACTs remain effective, continuous surveillance of transmission dynamics and drug effectiveness is essential to guide targeted and effective malaria control and elimination efforts in the region [26,46].

### 2.2. Study Design and Period

This observational cohort study, combined with an entomological experiment, was conducted from 1 January to 30 September 2025. Patients with microscopy-confirmed *P. falciparum* gametocyte infection who presented to the outpatient departments of the study sites were enrolled. Participants were allocated to either the AL-only group or the AL plus SLD-PQ group, based on primaquine availability at the time the clinician prescribed the treatment. Follow-up blood samples were collected on days 3, 7, 14, and 28 for parasitological assessments and mosquito MFAs.

### 2.3. Study Population and Inclusion/Exclusion Criteria

The study included patients who met the WHO criteria for evaluating and monitoring the efficacy of antimalarial drugs [47]. All individuals with suspected malaria who visited laboratory departments of the study sites for malaria diagnosis during the study period constituted the source population. The study population consisted of patients aged five and above, of both genders, who tested positive for *P. falciparum* mono-infection with gametocyte.

Eligibility criteria included the following: microscopically confirmed *P. falciparum* mono-infection with gametocyte; an axillary temperature ≥ 37.5 °C; the ability to swallow oral medication; permanent residency within the hospital’s catchment area; and willingness to comply with the study protocol. Patients were screened for eligibility. Additionally, participants age ≥ 5 years old who have confirmed uncomplicated *P. falciparum* mono-infection, show no signs of severe malaria symptoms and other severe co-morbid conditions, and show willingness to participate with informed consent were also part of the study. 

Exclusion criteria included infection with mixed or non-falciparum species confirmed by microscopy, pregnancy or breastfeeding, known hypersensitivity to the study drugs, signs of severe or complicated malaria, severe malnutrition, hemoglobin (Hgb) level below 5.0 g/dL, intake of AL within the previous 2 weeks, inability to take oral medication, continuous vomiting, and known chronic or severe diseases.

#### Operational Definitions

Gametocyte density is defined as the average number of gametocytes per unit of blood (typically per microliter) among malaria-infected individuals who were gametocyte-positive at diagnosis [48].

Severe malaria is a life-threatening clinical syndrome primarily caused by *P. falciparum*. It develops when asexual parasitemia results in vital organ dysfunction or high parasite biomass, and is marked by complications like cerebral malaria, severe anemia, respiratory distress, acute kidney injury, metabolic acidosis, hypoglycemia, and circulatory collapse (shock). Prompt diagnosis and immediate effective treatment are crucial to preventing death [49].

Malaria co-morbidity is a condition that significantly worsens the prognosis of malaria, such as HIV/AIDS, malnutrition, pregnancy, chronic diseases (e.g., diabetes or hypertension), or any immune-compromised states. These conditions increase susceptibility to malaria and can complicate its clinical management [50,51,52].

Oocyst infection rate: This is the proportion of *Anopheles* species that carry *Plasmodium* oocyst in their midgut during the time of examination [53,54].

### 2.4. Sample Size Determination

To compare gametocyte clearance between the AL plus SLD-PQ group and the AL-only group, we calculated the required sample size using a formula for comparing two independent proportions. This approach was guided by data from previous studies evaluating the efficacy of these regimens in reducing gametocyte prevalence and blocking transmission [12,55].

A systematic review and meta-analysis by Bassat Orellana et al. (2016) reported a gametocyte clearance rate of 83.3% in patients treated with AL alone based on a pre-treatment prevalence of 100% (498/498) and a post-treatment prevalence of 16.7% (83/498) [55]. In contrast, Mahamar et al. (2024) observed a 100% clearance rate in patients receiving AL combined with SLD-PQ, where all participants had detectable gametocytes before treatment (20/20) and none afterward [12].

Using these proportions, the following standard formula for comparing two independent proportions was applied:

$$n=\frac{{\left(Z\alpha /2+Z\beta \right)}^{2}\times [P1\left(1-P1\right)+P2(1-P2)]}{{\left(P1-P2\right)}^{2}}$$

where:

- *n* = required sample size per group;
- Zα/2 = 1.96 for a 95% confidence level;
- Zβ = 0.84 for 80% power;
- P1 = gametocyte prevalence reduction in the AL group = 0.833;
- P2 = gametocyte prevalence reduction in the AL + PQ group = 1.0.

Substituting these values provides the following:

$$\frac{{\left(1.96+0.84\right)}^{2}\times [0.833\left(1-0.833\right)+1.0\left(1-1.0\right)]}{(0.833-{1.0)}^{2}}=\frac{{\left(2.8\right)}^{2}\times 0.138}{0.02756}=\frac{7.84\times 0.138}{0.02756}=39.3$$

To account for the potential loss to follow-up or participant withdrawal, a 20% contingency was added [56]. This would result in a total enrollment requirement of 48 patients per group. This final number ensured adequate statistical power to detect a meaningful difference in gametocyte clearance and *Anopheles* mosquito infectivity between the two treatment arms, with 95% confidence and 80% power.

### 2.5. Sampling Technique and Data Collection

**Figure 2:**
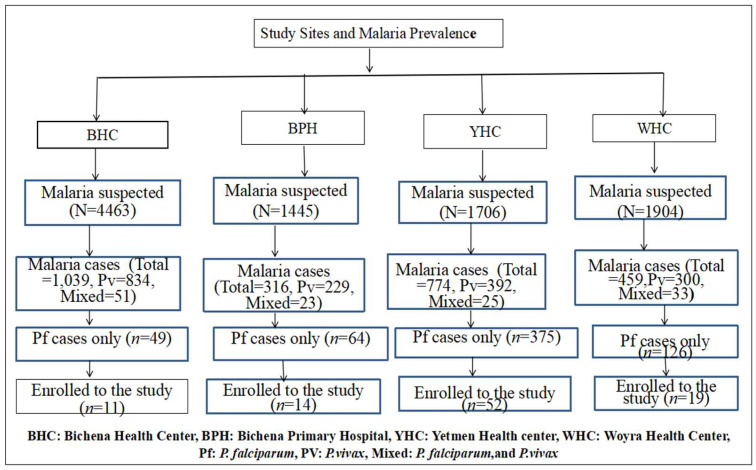
Number of confirmed malaria cases and sampling techniques employing consecutive sampling across study sites.

A passive case detection approach was used for recruitment. The sample was recruited from four institutions based on the prevalence of *P. falciparum* gametocyte detection (Figure 2). Patients with microscopically confirmed *P. falciparum* gametocyte carriage were recruited using a consecutive sampling technique after prescription of antimalarial drugs by the physician at the outpatient department of the study site. Participants were selected until the required sample size was achieved. Patients who met the study’s inclusion criteria and aligned with the study objectives were subsequently enrolled into the study. For each participant, socio-demographic and clinical information was documented, including baseline measurements of Hgb concentration (g/dL), axillary temperature (°C), and body weight (kg).

### 2.6. Parasitological Assessment

#### 2.6.1. Blood Film Examination

Gametocyte detection was performed using light microscopy (OLYMPUS CX22) by examining both thin (2 µL) and thick (6 µL) blood smears prepared from each sample on enrolment (day 0) and follow-up days 3, 7, 14, and 28. The smears were stained with 10% Giemsa and air-dried according to standard protocols to determine the presence or absence of *Plasmodium* species, as well as the quantification of gametocytes and asexual parasites [57].

Asexual parasite densities were determined against 200 white blood cells (WBCs) and gametocyte densities were also determined against 500 WBCs, assuming a standard mean leukocyte count of 800 WBCs per μL of blood [58], and converting the total number into parasites/µL (p/µL).

$$Asexual\;parasit\;density/µL=\frac{No.\;of\;Parasites\;counted}{No.\;of\;WBC\;counted}\times 8000\;WBCs/µL$$

$$Gametocyte\;density/µL=\frac{No.\;of\;gametocytes\;counted}{No.\;of\;WBCs\;counted}\times 8000\;WBCs/µL$$

Gametocyte density was classified according to the WHO malaria microscopy guidelines [59]. Based on counting the number of gametocytes in 1000 WBCs, gametocytemia was categorized as follows: low (1–10 gametocytes/μL), moderate (11–50 gametocytes/μL), and high (more than 50 gametocytes/μL).

At least two trained and experienced laboratory professionals verified blood smears before the results were reported to ensure the validity of blood film results. Discordant outcomes during malaria microscopy were defined as any instance in which the findings of two separate microscopists did not match. This included differences in parasite detection (positive versus negative), species identification (*P. falciparum*, *P. vivax*, or mixed infections), or estimates of parasite density that fell outside the acceptable range of variations. In these cases, a third, senior microscopist reviewed the slides to provide final, adjudicated result.

#### 2.6.2. Molecular Confirmation of Recurrent *Plasmodium* Infections

Submicroscopic co-infections and post-treatment clearance of *Plasmodium* species were assessed using the nested polymerase chain reaction (PCR) from dry blood spot (DBS) samples collected at enrollment (day 0) and on day 28 post treatment. Molecular analyses were conducted with a TC9639 Benchmark thermal cycler following the samples’ transport to the Tropical and Infectious Diseases Research Center (TIDRC) at Jimma University, Jimma, Ethiopia.

Genomic deoxyribonucleic acid (DNA) was extracted from DBSs using a modified Chelex-100 method (Wooden et al.) [60]. A 3 mm DBS punch was incubated overnight at 4 °C in 50 μL of 10% saponin and 950 μL of phosphate-buffered saline (PBS) mixture; this was followed by centrifugation and PBS washes. The dried pellets were treated with 150 μL of 20% Chelex^®^ 100 resin and distilled water, then incubated at 95 °C for 10 min with intermittent vortexing. After centrifugation (14,000 rpm for 10 min), the supernatant-containing parasite DNA was collected and stored at −20 °C. DNA concentration was measured via Nanodrop spectrophotometry.

##### Nested PCR Amplification

For the collected sample on the first day (day 0 DBS), the *P. vivax* (18S *ribosomal Ribonucleic Acid* (rRNA) gene) was amplified using a TC9639 thermal cycler to detect submicroscopic mixed infections. This was performed in a 25 µL reaction with 2 µL of template DNA, 0.4 µL rVIV1/rVIV2 primers [61,62], 6 µL PerfeCTa^®^ qPCR ToughMix^®^, and 16.2 µL of PCR grade water. The forward and reverse primer sequence of *P.vivax* was 5′ CGC TTC TAG CTT AAT CCA CAT AAC TGA TAC 3′ (rVIV1), and 5′ ACT TCC AAG CCG AAG CAA AGA AAG TCC TTA 3′ (rVIV2). The cycling conditions were as follows: 95 °C for 10 min; 30 cycles of 95 °C for 60 s; 58 °C for 60 s; 72 °C for 90 s; and a final extension at 72 °C for 10 min. The conditions were adopted from a previous study conducted elsewhere [63]. Positive (*P. vivax* gDNA) and negative controls were included.

Similarly, for the collected DBS sample on day 28, *P. falciparum* genomic DNA was amplified using 25 µL reaction with 2 µL of template DNA, 0.4 µL rFAL1/rFAL2 primers [61,62], and 6 µL PerfeCTa^®^ qPCR ToughMix^®^ and 16.2 µL PCR grade water to confirm parasite clearance. Cycling conditions were as follows: 95 °C for 10 min; 30 cycles of 95 °C for 60 s; 58 °C for 60 s; 72 °C for 90 s; and a final extension at 72 °C for 10 min. The forward and reverse primer sequence of *P*. *falciparum* was 5′ TTA AAC TGG TTT GGG AAA ACC AAA TAT ATT 3′ (rFAL1) and 5′ ACA CAA TGA ACT CAA TCA TGA CTA CCC GTC 3′ (rFAL2). Positive (*P. falciparum* gDNA) and negative controls were included.

##### Agarose Gel Electrophoresis

A 1.5% agarose gel in 1× TAE buffer was prepared, stained with Ethidium bromide, and cast with dual-size combs. After solidification, 10 µL of PCR products and molecular markers (100 bp ladder) were loaded. Electrophoresis was run at 90 voltages, and 400 amper for 75 min. DNA bands were visualized using an ultraviolet transilluminator and interpreted based on expected band sizes.

#### 2.6.3. Hematocrit Determination

In addition to these, blood samples were also used to assess treatment safety and tolerability of the drug was also monitored during the follow-up period. Hematocrit determination was also performed using hematocrit centrifuge (SH-120). Anti-coagulated blood, collected in a heparinized capillary tube, was centrifuged at 11,000 to 12,000 rpm for 5 min. The resulting hematocrit values were then measured using a micro-hematocrit reader, following the standard procedure outlined in previous protocols [64].

### 2.7. Treatment and Follow-Up

Drug administration was conducted according to the revised WHO guidelines [65], with dosing based on body weight. Treatment commenced on day 0 (the day of enrolment) for both groups: those receiving AL alone and those receiving AL in combination with an SLD-PQ. The AL tablets (20 mg artemether + 120 mg lumefantrine; batch: NAA24250A; Mfg: MNB/11/847; Exp: 04/2027; Himachal Pradesh, India) and the SLD-PQ (0.25 mg/kg; lot: 117327; MFG: 06/24; Exp: 06/26;Remedica Ltd., Limassol, Cyprus) were supplied by the Ethiopian Ministry of Health with support from WHO and administered in line with the National Malaria Treatment Guidelines [29].

All enrolled participants received the standard six-dose AL regimen, which was given twice daily over three consecutive days. The first dose of either AL alone or AL with SLD-PQ was administered under direct supervision at the study site. To facilitate follow-up, participants (or their parents/guardians) were issued appointment cards containing their name and identification code, with scheduled visits on days 3, 7, 14, and 28. Unscheduled visits were permitted for any participant who felt unwell during the follow-up period.

To ensure correct home administration of the remaining doses, clear verbal and written instructions were provided to patients and/or their parents or caretakers. Evening doses on days 0, 1, and 2 were to be taken 8 and 12 h, respectively, after the morning dose at home.

Participants were advised to take fatty foods or any type of meal immediately before or after taking the medication, in order to optimize drug absorption. Study participants took the tablets orally with a glass of water; for those unable to swallow tablets, the medication was crushed and administered after mixing with water. Adherence to the drug regimen and success of home administration were assessed during the follow-up visit on day 3.

At each scheduled visit, participants underwent interviews, and physical and clinical examinations, with particular attention given to danger signs or symptoms associated with malaria. Fever, adverse events, and parasitaemia were assessed using microscopy. At enrolment, molecular assays were conducted to detect submicroscopic mixed infections.

If a participant missed a scheduled visit, a designated home visitor traced them the same day and facilitated their referral to the Bichena Primary Hospital or the nearest health center. Any heparinized blood samples and clinical records obtained at local health facilities were promptly transported to the Bichena Primary Hospital under cold chain conditions to maintain sample integrity.

Participants who received AL alone were enrolled during periods when primaquine was unavailable at the study sites, despite it being prescribed by the attending physician. These individuals served as the control group, enabling comparison of gametocyte clearance and mosquito infectivity outcomes with those who received the combination therapy of AL plus SLD-PQ.

### 2.8. Study Participant Withdrawal

Participants lost to follow-up, infected with *P. vivax*, missing scheduled doses, or unwilling to continue to participate were excluded from the study.

### 2.9. Classification of Treatment Outcomes

Treatment outcomes were classified as early treatment failure (ETF), late clinical failure (LCF), late parasitological failure (LPF), and adequate clinical and parasitological response (ACPR) based on WHO guidelines [66].

### 2.10. Assessment of Adverse Events and Follow-Up

Adverse events (AEs) were evaluated through clinical and physical examinations and questioned using a standard list of AEs associated with malaria, AL alone, and AL plus SLD-PQ. Patients and caregivers were asked to report any unusual occurrences after drug administration, including the child’s tolerability to the treatment.

At each visit, participants provided an additional 2 mL of venous blood, which was used for thick and thin blood smears, DBS and MFA. Parasitemia and gametocyte densities were assessed through microscopy and PCR. In parallel, drug efficacy, safety, and adverse events were systematically evaluated.

Fever and adverse events were monitored using a digital thermometer, hematocrit levels were measured using a hematocrit centrifuge (SH-120), and potential side effects of SLD-PQ were assessed using the Hillman color scale. The principal investigator, in collaboration with trained laboratory personnel, oversaw the safety monitoring and laboratory evaluations. Significantly, laboratory staff were blinded to treatment group assignments to ensure unbiased assessment of outcomes.

DBS specimens from gametocyte carriers were stored at the Bichena Primary Hospital at temperatures below −20 °C until they were transported for PCR analyses. At the same time, fresh blood samples were used for membrane feeding.

### 2.11. Mosquito Infectivity Testing

**Figure 3:**
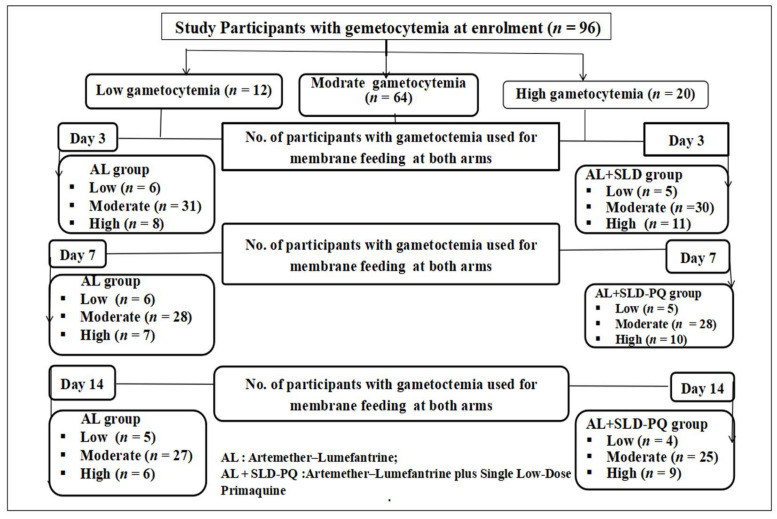
Number of participants with gametocytemia involved in membrane feeding assays during follow-up days (days 0, 3, 7, and 14) (the arrow denotes gametocytemia classification and follow-up time points).

Following gametocyte density classification, we assessed *Anopheles* mosquito infectivity by evaluating the proportion of infected mosquitoes after feeding on blood samples from participants grouped by gametocytemia level and treatment type (Figure 3). 

To evaluate infectivity, an average 25 *An. arabiensis* mosquitoes per treatment group were fed. Each experiment was conducted in duplicate to enhance the robustness of our findings. A total of 8166 *An. arabiensis* mosquitoes were dissected and analyzed across all groups.

### 2.12. Anopheles Mosquito-Rearing Membrane Feeding Assay

#### 2.12.1. Anopheles Mosquito Rearing

Eggs of *An.arabiensis* were obtained from the insectaries at the Aklilu Lemma Institute of Pathobiology, Addis Ababa University, and the TIDRC of Jimma University. They were then transported to the biology department’s insectary room at the Debre Markos University under conditions that maintained their viability. Upon arrival, the eggs were reared to adulthood under standard laboratory conditions. The rearing process was carried out at 27 ± 1.5 °C, with 80% relative humidity and a 12:12 light/dark cycle to ensure optimal development. The larvae were fed daily with yeast (*Saecchaomyces cerevisiae*, a product of Turkish Finising), and distilled water was added regularly to maintain water quality and volume. The pupae were carefully transferred to designated cages to facilitate safe emergence. Adult mosquitoes were maintained on a 10% sugar solution and fed non-*Plasmodium*-infected blood for colony upkeep. For MFAs, female *An. arabiensis* mosquitoes aged 3–5 days after emerging to adult from pupae were used, following a standardized protocol adapted from previous studies [67]. The *An. arabiensis* mosquitoes were starved for 12 h in order to facilitate the membrane feeding proccess.

#### 2.12.2. Anopheles Mosquito Membrane Feeding Assay and Dissection

MFAs were conducted using blood samples collected from gametocyte-positive individuals on day 0 (pre-treatment) and on days 3, 7, and 14 post treatment to assess changes in mosquito infectivity following treatment with AL or AL + PQ. For mosquito feeding, a 35 mm × 10 mm sized Petri dish was placed on a slide warmer. Then, 500 µL of infectious blood which carried the *P. falciparum* gametocyte sample was transferred to the dish and covered with a stretched parafilm membrane. Prior to placement on the mosquito cage, the dish was gently swirled to evenly distribute the blood, and then inverted so that the Parafilm side rested directly on the cage netting. A pre-warmed gel pack was placed on top of the Petri dish, to maintain the blood at physiological temperature (approximately 37 °C) and to stimulate mosquito feeding behavior; mosquitoes were allowed to feed for 30 min.

Once feeding was complete, engorged mosquitoes were maintained under standard insectary conditions. For each participant sample, two replicate batches of *An. arabiensis*, each containing at least 25 mosquitoes, were used. After feeding, engorged mosquitoes were separated and moved to new cages with access to a 10% sugar solution. They were maintained under controlled temperature and humidity conditions to support survival and parasite development. After 7 days, midgut dissections were performed to quantify the presence and density of oocysts, serving as a measure of transmission success and infectivity.

Oocysts were detected and quantified in infected mosquitoes by staining dissected midguts with 0.2% mercurochrome and examining them under a light microscope. The *An. arabiensis* mosquitoes midgut dissection was performed following WHO guidelines [68].

Female *An. arabiensis* mosquitoes were anesthetized with chloroform-soaked cotton wool. A drop of normal saline (0.85%) was placed on a clean slide, and the anesthetized mosquito was positioned ventral side up with its terminal segments centered in the normal saline drop. Using forceps on the thorax and a dissecting needle to gently pull the terminal abdominal segments, the midgut was carefully extracted.

The dissected midgut was then transferred to a new slide with a drop of 0.2% mercurochrome, covered with a clean coverslip, and examined using 10× times of a compound microscope for Oocyst visualization and counting as shown in Supplementary Figure S1.

### 2.13. Data Quality Control

All data collectors received pre-study training and were closely supervised during data collection. In addition, to minimize loss to follow-up, laboratory staff at health centers within the study catchment area were awarded to collect and process heparinized blood samples and to monitor participants for any adverse drug effects during scheduled visits at the site, rather than sending them to the hospital. Comprehensive quality control measures were applied across all laboratory procedures to ensure accuracy, reliability, and overall study integrity.

### 2.14. Outcomes

The study focused on two key outcomes. The primary outcome was gametocyte prevalence at specific time points (days 0, 3, 7, 14, and 28). The secondary outcomes were mosquito infection parameters assessed on days 3, 7, and 14 post treatment, evaluated via MFAs and Oocyst detection in mosquitoes dissected 7–8 days after membrane feeding. These outcomes were used to examine the association between gametocytemia and mosquito infectivity over time. Mosquito infectivity was quantified using three metrics: (1) the proportion of *P. falciparum*-infected participants whose blood infected at least one mosquito (infectious participants), (2) the percentage of mosquitoes harboring oocysts (infection rate), and (3) the mean number of oocytes per infected mosquito (oocytes density).

### 2.15. Data Management and Analysis

Data were coded, cleaned, and entered into SPSS version 25.0 for analysis. Prior to analysis, the dataset was checked for completeness and consistency. Microscopy sexual parasite (gametocyte) density was presented using mean per µL blood. Gametocyte prevalence at day 0, 3, 7, and 14 post treatment, human-to-mosquito infectious rate, and mosquito infection rate between the AL alone and AL + SLD-PQ groups were compared using descriptive statistics for age, sex, gametocytaemia, and sexual parasite density at baseline. Differences in baseline and post-treatment gametocyte density were tested between the two treatment groups using independent samples from *t*-test and within the group post treatment using paired sample *t*-test, respectively. The prevalence of gametocytes and infectious individuals were compared within and between treatment groups using two-sided Fisher’s exact tests (chi-square). The association between gametocyte density and mosquito infectivity was assessed using binary logistic regression; a *p*-value less than 0.05 was used as statistical significance value.

### 2.16. Ethics Approval and Consent to Participate

All procedures were conducted following the relevant guidelines and regulations. Ethical clearance for this study was granted on 30 December 2024, with reference number DMU/RTTD/75/10/2024, by the Institutional Research Ethics Review Committee of the Research and Technology Transfer Directorate of Debre Markos University. After explaining the study’s objectives and methodology, verbal consent was obtained from the participants or assent from legal protectors. Participants’ expenses were reimbursed for travel expenses incurred during study follow-up visits based on receipt. Patient confidentiality was strictly maintained throughout the data collection and analysis process.

## 3. Result

### 3.1. Baseline Characteristics, Enrollment, and Follow-Up of Study Participants

During the study period, 9518 individuals suspected of having malaria visited the laboratories at the study sites. Of these, malaria was confirmed in 2588 (27.2%) (95% CI: 26.3–28.1%) cases. *P. vivax* was responsible for 1755 (18.4%) (95%, CI: 17.66–19.22%) cases, and 132 (1.4%) (95%, CI: 1.15–1.62%) were mixed infections. The remaining 614 (6.4%) cases were due to *P. falciparum*, with 108 individuals confirmed by microscopy to carry gametocytes. From this group, 96 individuals with *P. falciparum* mono-infection and gametocyte positivity were enrolled in the cohort study. Of these, 48 participants received AL alone, while the other 48 received AL combined with an SLD-PQ. Three participants in the AL-only group and two participants in the AL-with-SLD-PQ group were confirmed to have mixed infections by nested PCR and were excluded retrospectively.

**Figure 4:**
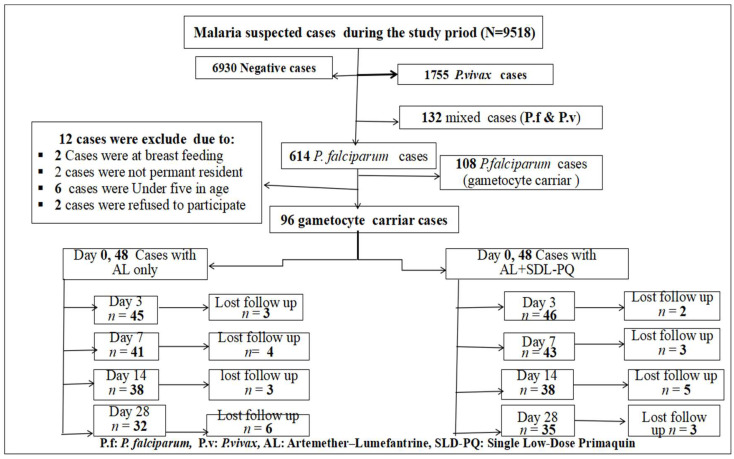
Number of participant at enrollment and follow-up flowchart for AL-only and AL + SLD-PQ treatment groups, from 1 January to 30 September 2025.

During the follow-up visits on days 3, 7, 14, and 28, the number of participants lost to follow-up were recorded as follows: three, four, three, and six in the AL-only group, and two, three, five, and three in the AL + SLD-PQ group, respectively. In total, 67 participants completed the therapeutic efficacy follow-up, as shown in Figure 4.

The summary of the participants’ baseline characteristics was similar across the study groups (Table 1).

### 3.2. Gametocyte Density and Prevalence Post Treatment

**Figure 5:**
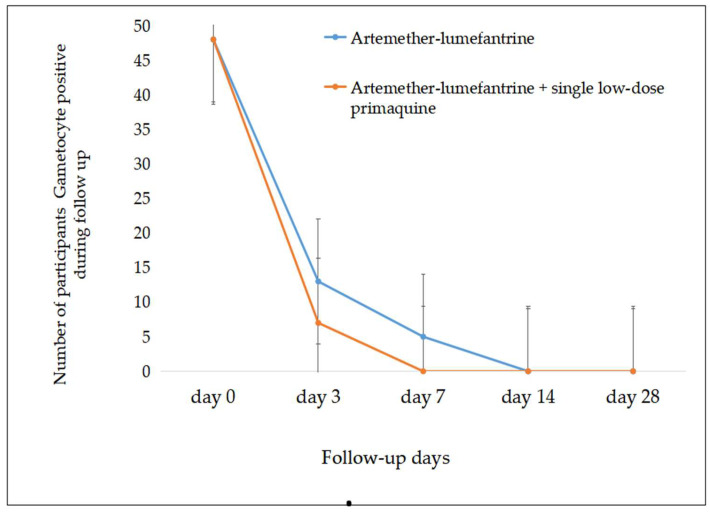
Rapid gametocyte prevalence reduction was observed post treatment in both groups (error bar showed standard error).

Although there was no significant difference in the participants’ gametocyte density between the two groups at baseline (t = 2.42, df = 88.5, *p* = 0.6), there was a statistically significant decline in gametocyte densities after initiation of treatment in both treatment groups. The gametocyte density decreased much faster in the AL-combined-with-SLD-PQ group than in AL-only group on day 3 (t = −3.5, df = 53.7, *p* = 0.001), day 7 (t = −2.21, df = 40, *p* = 0.033), and day 14 (*p* < 0.004). There was also a significant decline in gametocyte density post treatment in the AL-only group on day 3 (t = −8.2 df = 92, r = −0.6, *p* = 0.00), day 7 (t = −2.96, df = 88, r = −0.35, *p* = 0.004), and day 14 (t = 2.9, df = 89, r = −0.23, *p* = 0.005), and in the AL-with-SLD-PQ group on day 3 (t = −6.95, df = 93, r = −0.72, *p* < 0.001), day 7 (t = 2.74, df = 90, r = −0.27, *p* = 0.007), and day 14. A 60%, 87.8%, and 100% decline in gametocyte prevalence was observed on day 3, day 7, and day 14 of post treatment by AL alone and 84.8%, 100%, and 100% in the AL-with-SLD-PQ group, respectively. There was a significant difference for the mean of gametocyte density reduction in the groups, as well as between the groups (*p* = 0.004) (Table 2 and Figure 5).

#### Adverse Events and Recurrent Malaria

During enrollment, 87 participants received an intramuscular injection of diclofenac sodium (75 mg/3 mL; Reoung Pharmaceutical Co., Ltd., Zibo, China), and 96 participants were administered paracetamol (15 mg/kg; Micro Labs Limited, Mumbai, India) orally, as needed, for the management of fever and pain in addition to standard antimalarial treatment. No AEs were reported following the administration of either AL alone or AL in combination with SLD-PQ.

No participants withdrew from the study due to adverse events, and no events of special interest were reported. However, molecular analysis identified two cases of recurrent *P. falciparum* infection on day 28 (LPF). Although microscopy results were negative at that time point, PCR assays confirmed the presence of *P. falciparum* DNA in both individuals. These participants were from the AL-only treatment arm and were subsequently re-treated using AL + SLD-PQ.

### 3.3. Host Infectivity to An. arabiensis Mosquitoes

Before starting treatment, MFAs were conducted using blood samples from 88 participants. A total of 2400 *An. arabiensis* mosquitoes were exposed, and 1848 were successfully fed. Among the participants, 63.6% (56/88) had at least one mosquito infected. The oocyst density in infected mosquitoes ranged from 3 to 324 per midgut.

On day 3 post treatment, blood samples were collected from 91 participants and MFAs were completed for 86 individuals (42 from the AL-only arm and 44 from the AL + SLD-PQ arm). A total of 4300 *An. arabiensis* mosquitoes were exposed to the blood meals, of which 1978 were fully engorged and subsequently dissected: 961 (48.6%) from the AL-only group and 1017 (51.4%) from the AL + SLD-PQ group. The proportion of participants whose samples resulted in at least one infected mosquito was statistically significantly higher in the AL-only arm (12/42; 28.6%) compared to the AL + SLD-PQ arm (3/44; 6.8%) on day 3 (*p* < 0.001). Oocyst density in infected mosquitoes ranged from 3 to 296 in the AL-only group and from 3 to 179 in the AL + SLD-PQ group (*p* = 0.001). Among the infectious individuals, seven in the AL-only group and two in the AL + SLD-PQ group were microscopically gametocyte-negative on day 3, indicating the presence of submicroscopic gametocytemia capable of sustaining transmission.

On day 7 post treatment, MFAs were conducted on 41 participants from the AL-only group and 42 from the AL + SLD-PQ group. A total of 4150 *An. arabiensis* mosquitoes were exposed, with 1066 fully engorged and dissected from the AL-only group and 985 from the AL + SLD-PQ group. The AL-only group continued to show a higher proportion of infectious samples, with 3 out of 41 participants (7.3%) resulting in at least one infected mosquito, and oocyst densities ranging from 2 to 186 per midgut. In contrast, no infections were detected in mosquitoes fed on blood from participants in the AL + SLD-PQ group (0/42; 0%) (*p* < 0.001). Notably, two of the three infectious individuals in the AL-only group were microscopically gametocyte-negative on day 7, suggesting ongoing submicroscopic transmission potential.

**Figure 6:**
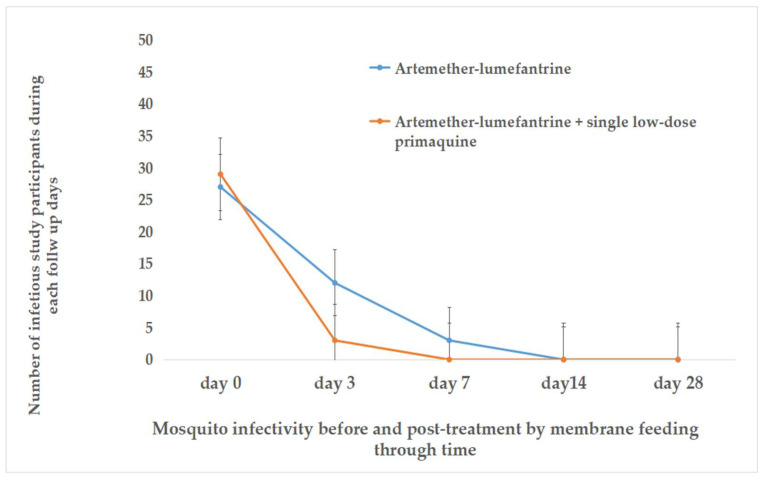
The relationship between gametocyte carriage and *An. arabiensis* mosquito infectivity pre and post treatment.

**Figure 7:**
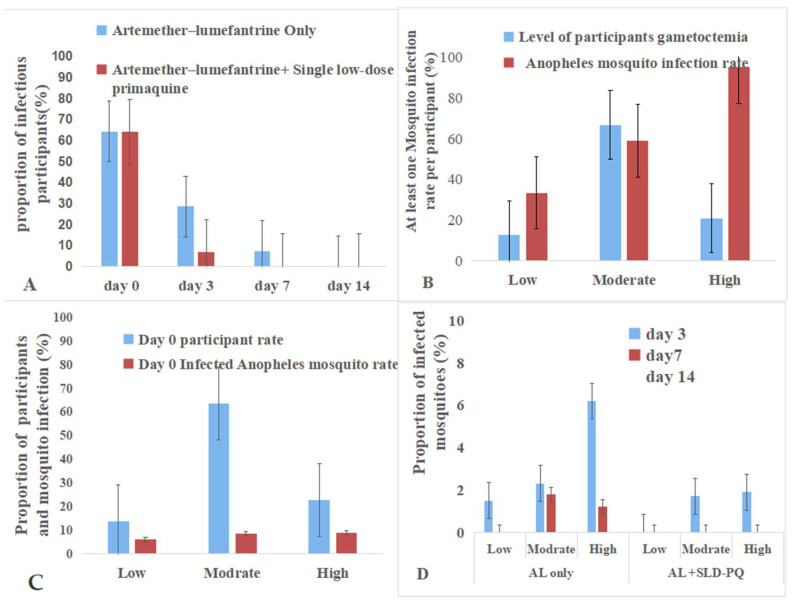
Gametocyte density and infectivity to *An.arabiesis* mosquitoes. (**A**): The addition of SLD-PQ accelerates transmission blocking, eliminating infectiousness earlier than AL alone. (**B**): The pattern highlights that individuals with higher gametocytemia are the major drivers of onward malaria transmission at baseline. (**C**): This indicates that while gametocyte carriers were common, only a small fraction contributed substantially to mosquito infection at baseline. (**D**): It showed that mosquito infectivity decreases over time in both treatment arms and across all gametocyte density categories.

By day 14 post treatment, blood samples were collected from 38 participants in the AL-only group and 38 in the AL + SLD-PQ group for MFAs. A total of 3150 *An. arabiensis* mosquitoes were exposed, with 1068 fully engorged and dissected from the AL-only group and 1221 from the AL + SLD-PQ group. At this point, no infected mosquitoes were detected in either treatment group, indicating a complete loss of transmissibility by day 14 (Table 3, Figure 6 and Figure 7).

### 3.4. Gametocyte Density and Infectivity to An. arabiensis Mosquitoes

These findings highlight the strong link between gametocyte density and transmission potential and demonstrate the enhanced efficacy of adding SLD-PQ to AL in rapidly reducing post-treatment infectivity (Table 4 and Figure 7). At enrollment, gametocyte densities among participants were low in 12.5% (12/96), moderate in 66.7% (64/96), and high in 20.8% (20/96). The infectivity to *An. arabiensis* associated with gametocyte density is as follows: 95% of high, 58.9% of moderate, and 33.3% of low gametocytemia samples infected at least one mosquito. Participants with high gametocytemia were 2.46 times more likely to infect mosquitoes compared to those with low gametocytemia (OR = 2.46, 95% CI: 1.5–2.90, *p* = 0.04),

Post treatment, mosquito infectivity declined more rapidly in the AL + SLD-PQ group than in the AL-only group. By day 3, infectivity persisted in the AL-only arm across all gametocyte densities, whereas the AL + SLD-PQ arm showed marked reduction. By day 7, AL-only participants still transmitted at low rates, while the AL + SLD-PQ arm completely blocked transmission. By day 14, both treatment arms showed no mosquito infection, despite detectable gametocytemia in some participants (Table 4).

## 4. Discussion

This study provided compelling evidence on gametocyte dynamics and infectivity to *An. arabiensis* mosquitoes, emphasizing the comparative efficacy of AL alone versus AL combined with SLD-PQ. At enrollment, baseline gametocyte densities were similar across treatment groups, ensuring a balanced comparison of treatment effects. Supporting this, AL also reduces gametocyte carriage and mosquito infection rates more effectively than newer ACTs such as dihydroartemisinin–piperaquine (DP) [69]. However, the duration of carriage varies by regimen [36,70,71]. Others also have shown that prompt treatment can limit the spread of resistant parasites, emphasizing the need for timely intervention [72]. The observed differences in gametocyte dynamics suggest that treatment strategies must be tailored to minimize transmission potential.

After starting treatment, our findings showed a significantly faster reduction in gametocyte prevalence and density with the combination of AL and SLD-PQ compared to AL alone. By day 3, prevalence dropped by 84.8% in the AL-and-SLD-PQ group versus 60% in the AL-only group, with complete clearance achieved by day 7 in the combination group and by day 14 in the AL-only group. These results align with other studies indicating that adding SLD-PQ accelerates gametocyte clearance and reduces infectivity [15,36,55,73,74]. In contrast, non-artemisinin regimens like SP plus amodiaquine have been associated with prolonged gametocyte carriage and higher post-treatment transmission [75]. The study provides strong evidence for the efficacy of AL combined with SLD-PQ in reducing gametocytes and its impact on malaria transmission.

This study confirms that AL alone significantly reduces gametocyte carriage and infectivity, while the addition of SLD-PQ greatly enhances clearance rates, shortens the infectious period, and achieves complete transmission interruption by day 7. Supporting this, other reports have indicated that AL treatment results in a significant reduction in gametocyte prevalence [71,76]. Similar findings have shown that AL alone reduces gametocyte prevalence, although it does not totally eliminate the risk of transmission [70,77]. In contrast, non-artemisinin-based regimens, such as SP, have been linked to increased or prolonged gametocyte carriage. This further underscores the importance of ACTs and particularly the adjunct use of SLD-PQ in effective gametocyte clearance and transmission reduction [78,79]. It is also crucial to consider the role of environmental factors and genetic markers in gametocyte production, which may further complicate elimination strategies [80]. These findings are in line with WHO recommendations that advocate for SLD-PQ as a gametocytocidal adjunct to ACT, highlighting its potential role in malaria elimination strategies, particularly in regions like Ethiopia, where *An. arabiensis* is the dominant vector, and reducing transmission is a national priority.

During the baseline MFAs, a density-dependent relationship between gametocyte burden and mosquito infectivity was observed: 95% of high-density carriers infected mosquitoes, compared to 59% and 33% of moderate- and low-density carriers, respectively. This pattern aligns with evidence showing that gametocyte density correlates positively with transmission success and oocyst density in mosquitoes [81,82,83]. Consistent with previous studies, high-density gametocytemia remains the strongest predictor of transmission; even low-density carriers significantly contribute to the ongoing malaria spread [81,82,83,84,85,86,87]. Furthermore, sex-specific gametocyte dynamics and host genetic factors, such as hemoglobinopathies, have also been reported to affect infectivity [81,85,88,89,90,91]. Additionally, individuals with a higher prevalence of gametocytes may increase transmission potential due to the multiplicity of infection [89]. Together, these complexities highlight the importance of targeting both symptomatic and asymptomatic carriers in elimination programs.

Mosquito feeding assays further confirmed the enhanced transmission-blocking activity of SLD-PQ. On day 3, only 6.8% of participants in the AL-combined-with-SLD-PQ group remained infectious compared to 28.6% in the AL-only group (*p* = 0.001). By day 7, none of the participants receiving AL combined with SLD-PQ infected the mosquitoes, whereas residual infectivity persisted in the AL-only group. These findings align with previous trials demonstrating the superior efficacy of SLD-PQ in suppressing submicroscopic gametocytemia and reducing post-treatment transmission potential [3,55,76,92,93,94]. Significantly, SLD-PQ was effective across all baseline density strata, eliminating infectivity even among high-density carriers.

Other studies reported that non-artemisinin combination therapies result in a prolonged gametocyte carriage post-treatment [36,76,95], while ACT reduces this duration to about 13.4 days. The addition of primaquine can further decrease this duration fourfold [96]. While other infections lasting three months or longer show nearly universal gametocyte presence, shorter infections often result in low-density gametocyte carriage, limiting their transmission potential [96]. The implication is clear: adding SLD-PQ can shorten the infectious window, which is crucial in epidemic settings or areas aiming for local elimination, and regions threatened by artemisinin resistance, potentially helping to slow the emergence and spread of drug-resistant parasites. This approach is also supported by strategies such as mass drug administration and transmission-blocking vaccines, which can be optimized by focusing on individuals with longer gametocyte carriage [97].

Safety is a crucial factor for the implementation of SLD-PQ. In our study, no serious AEs were reported but headaches and dry cough were presented and resolved without any complications related to the treatment. These findings support the tolerability of SLD-PQ. Notably, recrudescent infections occurred only in the AL-only group, indicating that AL combined with SLD-PQ may also help suppress submicroscopic parasitemia. Studies have confirmed that SLD-PQ is linked to a temporary reduction in hemoglobin levels in G6PD-deficient individuals, but serious adverse hematological events were rare [98,99,100].

## 5. Limitations and Operational Considerations of the Study

A potential limitation of this study is that the allocation to the AL-plus-SLD-PQ group versus the AL arm was not randomized, but determined by the intermittent stock availability of primaquine in the study area. In addition, only 67 of the 78 participants estimated by the sample size calculation completed the follow-up for gametocyte clearance assessment, which may have introduced limitations. By day 14, which is the final time point for evaluating mosquito infection, 76 participants remained under follow-up, thereby reducing the potential effect of this shortfall on study findings. One limitation was that the presence and absence of gametocytes could not be detected by molecular methods post treatment on days 3, 7, and 14. Another limitation is the lack of molecular gametocyte quantification, which affects the ability to establish a clear relationship between low gametocyte densities and infectiousness. Consequently, female and male gametocytes were not assessed, which restricted the ability to fully understand gametocyte dynamics and their possible role in transmission. Similarly, another limitation is that the mosquito feeding experiments were performed using a Petri dish gel warmer, which may have reduced mosquito infectivity and active mosquito feeding, resulting in variability both within and between experiments. This may have complicated the estimation of the drug effect and should be taken into account in the analysis to interpret the results better.

Additionally, since oocyst detection was also achieved using microcopy, sub-microscopy infections arising from gametocytes exposed to antimalarial drugs may have been missed. This study, while robust, does have limitations. Moreover, while MFAs are considered the gold standard for assessing infectivity, they may not fully reflect natural transmission dynamics in the field. Nonetheless, the use of large mosquito numbers, standardized protocols, and consistent trends across multiple time points strengthens confidence in the findings.

## 6. Implications for Malaria Elimination

The findings strongly advocate for the operational integration of SLD-PQ into national treatment policies for *P. falciparum* malaria, particularly in regions with persistent transmission. The marked and rapid reduction in gametocyte carriage and mosquito infectivity following AL combined with SLD-PQ underscores its potential to interrupt local transmission chains, which is a cornerstone of malaria elimination strategies. While targeting gametocyte carriage duration is crucial, the variability in individual responses to treatment and the emergence of drug resistance may complicate the effectiveness of these strategies, necessitating ongoing research and adaptation of control measures.

## 7. Conclusions and Recommendations

This study highlights the increased effectiveness of combining AL with an SLD-PQ in reducing gametocyte prevalence and interrupting malaria transmission. While AL alone showed significant gametocyte clearance by day 14, the addition of SLD-PQ accelerated this reduction, achieving 100% clearance by day 7. Notably, the transmission potential to *An. arabiensis* mosquitoes was significantly reduced in the AL + SLD-PQ group, with complete suppression of infectivity by day 7, even among individuals with high baseline gametocytemia. In contrast, the AL-only group maintained a measurable though decreasing transmission potential up to day 7. The study also reaffirms the strong positive correlation between gametocyte density and mosquito infectivity, while highlighting that even submicroscopic gametocytemia can sustain transmission. This finding underscores the limitations of relying solely on microscopy for transmission surveillance and emphasizes the need for sensitive molecular tools. Both treatment regimens had minimal, short-lived, and manageable adverse events, supporting the safety profile of SLD-PQ when used as part of supervised malaria case management.

Based on the findings, the routine integration of SLD-PQ with AL for *P. falciparum* treatment should be prioritized, especially in settings focused on elimination, due to its clear benefit in swiftly reducing gametocyte carriage and mosquito infectivity. National malaria control programs are encouraged to adopt molecular diagnostic tools, like quantitative real time PCR, to detect submicroscopic gametocytemia better and improve infectious reservoir surveillance. Individuals with high gametocytemia should be prioritized for targeted interventions, including enhanced vector control measures and systematic follow-up, to reduce malaria transmission potential. Further research is needed to evaluate the safety and effectiveness of AL + SLD-PQ in special populations such as pregnant women and children. Additionally, assessing the broader community-level impact of this regimen on transmission dynamics will help inform scalable policy decisions. Finally, training frontline health workers in proper administration, monitoring for adverse effects, and ensuring follow-up is essential for maximizing the therapeutic and transmission-blocking potential of SLD-PQ.

## Figures and Tables

**Table 1:** Summary of participant baseline characteristics between the study groups.

Characteristics	AL Only (*n* = 48)	AL + SLD-PQ (*n* = 48)
Male	33 (68.8%)	31 (64.6)
Female	15 (31.3%)	17 (35.4)
Median age in year (IQR)	24 (19.2–33.0)	27 (20.2–40.0)
Median weight in kg (IQR)	50.6 (45.9–58.2)	49.6 (43.2–55.4)
Median hemoglobin in g/dL (IQR)	13.0 (12–15)	13.1 (11.6–14.0)
Median axillary temperature in °C (IQR)	38.6 (37.9–39.4)	38.3 (37.9–38.7)
Median asexual parasitaemia density per µL (IQR)	2720.0 (960–4960)	2560.0 (1760.0–4860.0)
Mean gametocyte density per µL (±std)	32.6 (22.1)	35.0(22.6)

AL: Artemether–Lumefantrine, SLD-PQ: single low-dose primaquine, IQR: interquartile range, std: standard deviation, kg: kilogram, °C: degree Celsius.

**Table 2:** Gametocyte prevalence during follow-up and reduction post treatment.

Treatments		Gametocyte Prevalence (%)	Mean Gametocyte Density (±std)/µL	Reduction in Gametocyte Prevalence	Within-Group (Mean ± std)	*p* Value	Between-Group (Mean diff)	*p* Value
Day 0 (Pre treatment)	AL alone	48/48 (100%)	32.6 (22.1)	-	-	-	-	Ref
	AL + SLD-PQ	48/48 (100%)	35. 0(22.6)	-	-	-	−2.42	0.6
Day 3	AL alone	18/45 (28.9%)	6.9 (9.8)	27/45 (60%)	−18.3 (21.7)	<0.001	-	Ref
	AL + SLD-PQ	7/46 (15.2%)	1.4 (3.5)	39/46 (84.8%)	−17.1 (23.8)	<0.001	−5.4	0.001
Day 7	AL alone	5/41 (12.2%)	1.3 (4.2)	36/41 (87.8%)	2.6 (8.1)	0.004	-	Ref
	AL + SLD-PQ	0/43 (0%)	0	43/43 (100%)	0.8 (2.8)	0.007	−1.4	0.033
Day 14	AL alone	0/38 (0%)	0	38/38 (100%)	0.9 (2.9)	0.005	-	
	AL + SLD-PQ	0/38 (0%)	0	30/38 (100)	-	-	-	
Day 28	Al alone	0/32 (0%)	0	32/32 (100%)	-		-	
	AL + SLD-PQ	0/35 (0%)	0	35/35 (100%)	-		-	

AL: Artemether–Lumefantrine, SLD-PQ: single low-dose primaquine, std: standard deviation.

**Table 3:** Mosquito infectivity before and after treatment.

Treatments		Infectious Individuals	Mosquito Infection Rate	Reduction in Mosquito Infection Rate	Within-Group	Between-Group	*p*-Value
Day 0 (Pre treatment)	AL alone	27/42 (64.3%)	69/879 (7.9%)	-	-	-	-
	AL + SLD-PQ	29/46 (64%)	83/969 (8.6%)	-	-	-	-
Day 3	AL alone	12/42 (28.6%)	28/961 (2.9%)	62.9%	0.02		Ref
	AL + SLD-PQ	3/44 (6.8%)	0/1017 (0%)	100%	0.00	100%	0.00
Day 7	AL alone	3/41 (7.3%)	11/1066 (1.0%)	86.9%	0.00		Ref
	AL + SLD-PQ	0/43 (0%)	0/985 (0%)	100%	0.00	100%	0.01
Day 14	AL alone	0/38 (0%)	0/1068 (0%)	100%	0.00	Ref	-
	AL + SLD-PQ	0/38 (0%)	0/1221 (0%)	100%	0.00	(0)%	

Individuals were classed as infectious if DMFAs resulted in at least one mosquito with any number of oocytes.

**Table 4:** Association between gametocyte density at enrollment and *An. arabiensis* mosquito infectivity following membrane feeding assays.

	Gametocytemia Classification	No. of Participants Who Used Blood for Membrane Feeding Assay	No. of *An. arabiensis* Used for Membrane Feeding	No. of Infected *An. arabiensis* (%)	Oocyst Density in Rang	Association b/n Gametocyte Density and Mosquito Infectivity	
						Odd Ratio (95%, CI)	*p* Value
Base line	Low	12	252	15 (5.9%)	3–154	ref	
	Moderate	56	1160	98 (8.4%)	6–211	1.46 (0.82–2.60)	0.19
	High	20	436	39 (8.9%)	4–324	2.46 (1.5–2.90)	0.04
	Total	88	1848	152 (8.2%)	-	-	
Day 3 Al only	Low	6	137	2 (1.5%)	3–48	ref	
	Moderate	31	646	15 (2.3%)	3–296	1.55 (0.32–7.47)	0.58
	High	8	178	11 (6.2%)	3–211	4.41 (1.9–21.3)	0.04
	Total	42	961	28 (2.9%)	-	-	-
Day 3 AL + SLD-PQ	Low	5	123	0	-	-	-
	Moderate	28	632	11 (1.7%)	3–167	-	-
	High	11	262	5 (1.9%)	3–179	-	-
	Total	44	1017	16 (1.6%)	-	-	-
Day 7 AL only	Low	6	160	0	-	-	-
	Moderate	28	729	13 (1.8)	2–186	-	-
	High	7	167	2 (1.2%)	1–32	-	-
	Total	41	1066	15 (1.4%)	-	-	-
Day 7 AL + SLD-PQ	Low	5	152	0	-		
	Moderate	28	587	0	-	-	-
	High	10	246	0	-	-	-
	Total	43	985	0	-	-	-
Day 14 AL only	Low	5	147	0	-	-	
	Moderate	27	748	0	-	-	-
	High	6	173	0	-	-	-
	Total	38	1068	0	-	-	-
Day 14 AL + SLD-PQ	Low	4	131	0	-		
	Moderate	25	826	0	-	-	-
	High	9	264	0	-	-	-
	Total	38	1221	0-	-	-	-

AL: Artemether–Lumefantrine, SLD-PQ: single low-dose primaquine, CI: confidence interval.

## Data Availability

The original contributions presented in this study are included in the article. For further inquiries, please contact the corresponding author.

## Supplementary Materials

The following supporting information can be downloaded at https://www.mdpi.com/article/10.3390/tropicalmed11010019/s1; Figure S1: Oocysts stained with mercurochrome and observed under a 10× objective after dissecting the midgut of membrane-fed *Anopheles arabiensis*.

## Abbreviations

ACPR: adequate clinical and parasitological response, ACT: artemisinin-based combination therapies, AEs: adverse events, AL: artemether–lumefantrine, CI: confidence interval, DBS: dried blood spots, DNA: *deoxyribonucleic acid*, DP: dihydroartemisinin–piperaquine, FMOH: Federal Ministry of Health, ETF: early treatment failure, G6PD: Glucose-6-phosphate dehydrogenase deficiency, Hgb: hemoglobin, LCF: late clinical failure, LPF: late parasitological failure, MFAs: membrane feeding assays, SLD-PQ: single low-dose primaquine, PBS: phosphate buffer saline, PCR: polymerase chain reaction, rRNA: ribosomal ribonucleic acid, SP: sulfadoxine–pyrimethamine, TIDRC: Tropical and Infectious Diseases Research Center, WBCs: white blood cells, WHO: World Health Organization.
