# Supplementary material for: The Effect of Artemether–Lumefantrine Combined with a Single Dose of Primaquine on Plasmodium falciparum Gametocyte Clearance and Post-Treatment Infectivity to Anopheles arabiensis
Source: Trop Med Infect Dis. 2026 Jan 8;11(1):19. doi: 10.3390/tropicalmed11010019 (PMC12846533; doi:10.3390/tropicalmed11010019)
Supplement: Supplementary file 1 [file tropicalmed-11-00019-s001.zip › tropicalmed-3976414-supplementary.pdf]

Tropical Medicine and Infectious Disease

MDPI

Supplementary Materials

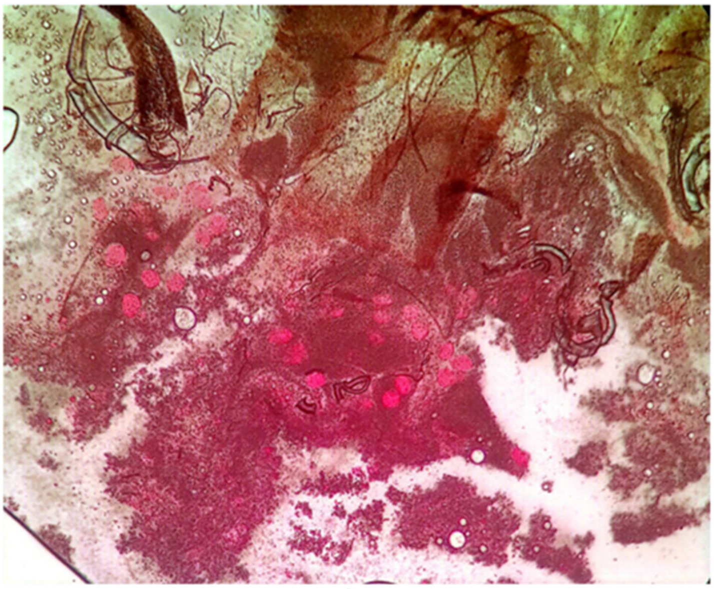

Figure S1. Oocysts stained with mercurochrome and observed under a 10× objective after dissecting the midgut of membrane-fed *Anopheles arabiensis*.

Disclaimer/Publisher's Note: The statements, opinions and data contained in all publications are solely those of the individual author(s) and contributor(s) and not of MDPI and/or the editor(s). MDPI and/or the editor(s) disclaim responsibility for any injury to people or property resulting from any ideas, methods, instructions or products referred to in the content.
